# SWAN: Subset-quantile Within Array Normalization for Illumina Infinium HumanMethylation450 BeadChips

**DOI:** 10.1186/gb-2012-13-6-r44

**Published:** 2012-06-15

**Authors:** Jovana Maksimovic, Lavinia Gordon, Alicia Oshlack

**Affiliations:** 1 Murdoch Childrens Research Institute, 50 Flemington Road, Parkville 3052, Australia

## Abstract

DNA methylation is the most widely studied epigenetic mark and is known to be essential to normal development and frequently disrupted in disease. The Illumina HumanMethylation450 BeadChip assays the methylation status of CpGs at 485,577 sites across the genome. Here we present Subset-quantile Within Array Normalization (SWAN), a new method that substantially improves the results from this platform by reducing technical variation within and between arrays. SWAN is available in the *minfi *Bioconductor package.

## Background

DNA methylation, which is the addition of a methyl group to the cytosine of a CpG dinucleotide, is one of the most widely studied epigenetic modifications in human development and disease. Changes in DNA methylation are vital for normal development and differentiation [1], whilst aberrant methylation is involved in diseases such as diabetes, schizophrenia, multiple sclerosis and cancer [2-4]. As interest in epigenetics, and particularly DNA methylation, has increased, analysis methods have had to evolve in scale and resolution. Currently, several microarray and next-generation sequencing technologies are available that allow the interrogation of DNA methylation on a genome-wide scale [5-14]. Each of these approaches has inherent strengths and weaknesses, which have been compared and discussed in several recent reviews [15-18]. As sequencing-based DNA methylation assays become more affordable, it is anticipated that they will be more widely used in this arena; at present, however, they are still too costly for most studies, particularly those that involve large numbers of samples. Consequently, methylation arrays are a popular alternative for high-throughput DNA methylation analyses.

**Figure 1:**
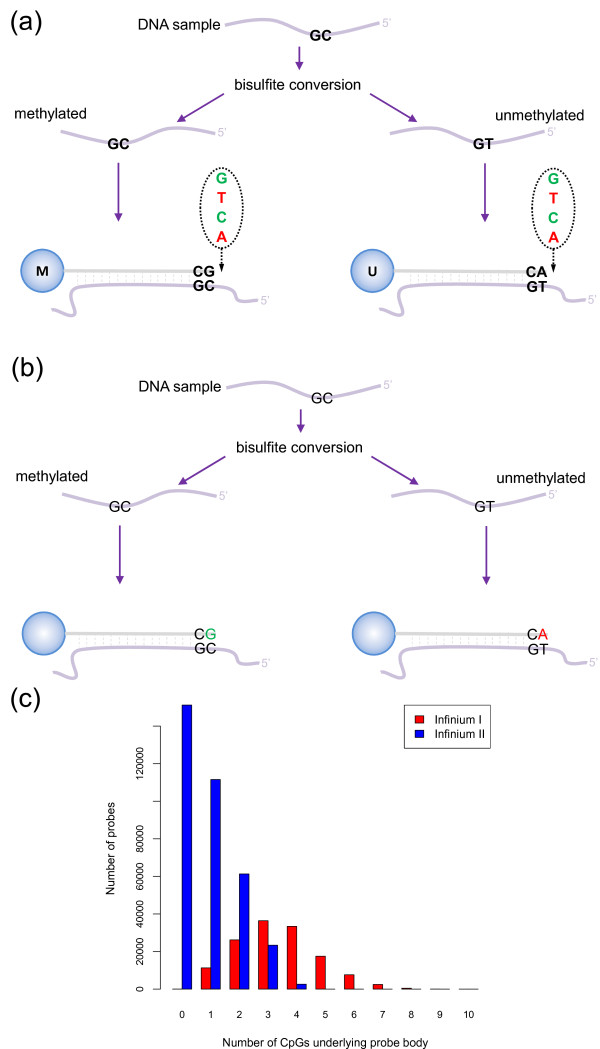
**Illumina Infinium HumanMethylation450 assay**. **(a) **Infinium I assay. Each individual CpG is interrogated using two bead types: methylated (M) and unmethylated (U). The probe design assumes that all CpGs underlying the probe body have the same methylation status as the target CpG. Both bead types will incorporate the same labeled nucleotide for the same target CpG, thereby producing the same color fluorescence. The nucleotide that is added is determined by the base downstream of the 'C' of the target CpG. The proportion of methylation, β, can be calculated by comparing the intensities from the two different probes in the same color: β= M/(U + M). **(b) **Infinium II assay. Each target CpG is interrogated using a single bead type. A probe may have up to three underlying CpG sites, with a degenerate R base corresponding to the 'C' of each CpG. Methylation state is detected by single base extension at the position of the 'C' of the target CpG, which always results in the addition of a labeled 'G' or 'A' nucleotide, complementary to either the 'methylated' C or 'unmethylated' T, respectively. Each locus is detected in two colors, and methylation status is determined by comparing the two colors from the one position: β = Green (M)/(Red (U) + Green (M)). **(c) **The number of CpG dinucleotides in the body of the probe according to Infinium probe type. Infinium I probes have significantly more CpGs in the probe body.

DNA methylation profiling using Illumina's Infinium technology was first utilized on the Infinium HumanMethylation27 (27k) BeadChip [12,19]. More recently, the genomic coverage of the array was dramatically increased, leading to the production of the Infinium HumanMethylation450 (450k) BeadChip, which interrogates the methylation status of 485,577 CpGs in the human genome. The Infinium assay detects methylation status with single base resolution, without the need for methylated DNA capture, thereby avoiding capture-associated biases. The 50 bp Infinium methylation probes query a [C/T] polymorphism created by bisulfite conversion of unmethylated cytosines in the genome. However, the Infinium 450k methylation platform is unique in that it uses a combination of two distinct probe types, Infinium I and II (Figure 1a,b).

The Infinium I design, which was previously employed on the 27k arrays, uses fluorescence from two different probes, unmethylated (converted) and methylated (unconverted), to assess the level of methylation of a target CpG. If a target CpG was methylated in the sample, the DNA fragment will remain unconverted after bisulfite treatment and will therefore bind to the complementary 'methylated' probe, which terminates at the 3' end with a cytosine. If the target CpG was unmethylated, however, binding will occur to the complementary 'unmethylated' probe, which terminates at the 3' end with a thymine. Binding at either probe is followed by single base extension that results in the addition of a fluorescently labeled nucleotide (Figure 1a). It is assumed that the methylation status of CpGs underlying the 50 bp probe body is correlated to that of the target CpG such that CpGs in the probe body of an unmethylated (converted) probe are also converted, while CpGs in the body of a methylated (unconverted) probe will remain unconverted. By contrast, the Infinium II design uses only a single probe per target CpG, which incorporates a 'degenerate' R-base at any underlying CpG sites in the probe body. The 3' end of each Infinium II probe is complementary to the base directly upstream of the 'C' of the target CpG. Methylation state is detected by single base extension at the position of the 'C' of the target CpG, which always results in the addition of a labeled 'G' or 'A' nucleotide, complementary to either the 'methylated' C or 'unmethylated' T, respectively (Figure 1b).

The Infinium II design is the preferred probe design for the 450k chip. Bibikova *et al*. [20] demonstrated that the Infinium II probes could have up to three CpG sites underlying their 50 bp probe body without affecting data quality. However, hybridization kinetics and specificity were often compromised in regions of higher CpG density and therefore Infinium I probes are still used to expand the number of CpG sites that can be assayed. Consequently, many of the Infinium I probes contain three or more underlying CpGs, whilst most Infinium II probes have less than three underlying CpGs (Figure 1c).

Technical differences between the Infinium I and Infinium II probe types have been observed. Bibikova *et al*. [20] noted a difference in the β value distributions they produced, where β is defined as the proportion of the total signal coming from the methylated channel. Specifically, they noticed a compression in the β value distribution of Infinium II probes compared to Infinium I. Similarly, Dedeurwaerder *et al*. [21] reported that the β values obtained from the Infinium II probes displayed a narrower range than those obtained from Infinium I probes and suggested that Infinium II probes are less sensitive for the detection of extreme methylation values due to the two-color detection method used. They suggested a simple scaling of β values for Infinium II probes and reported improved results in terms of validation against bisulfite pyrosequencing data, but also noted potential difficulties in applying this procedure to cancer samples.

Here we present a novel method to normalize between Infinium probe types on the 450k platform. This method derives from normalization methods that have been hugely successful for microarray expression platforms [22-24]. Specifically, we introduce a Subset-quantile Within Array Normalization (SWAN) method that allows the Infinium I and II probes within a single array to be normalized together. We show that this method substantially reduces the differences in β value distribution observed between Infinium I and II probes. We also demonstrate that this method improves correlation between technical replicates, whilst increasing the number of significantly differentially methylated probes that are detected. SWAN is written in the R programming language and is available in the *minfi *package [25] from Bioconductor.

## Results

### Subset-quantile Within Array Normalization

**Figure 2:**
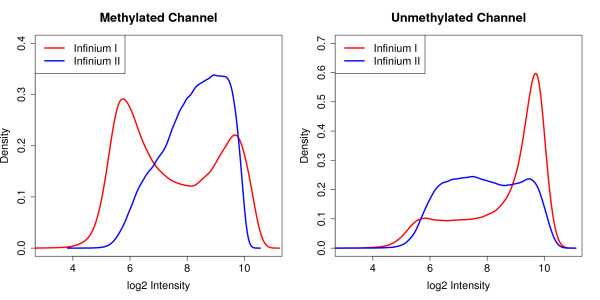
**Intensity distributions of the Infinium I and II probe types in the methylated and unmethylated channels for normal human kidney sample TCGA-B0-5092-11**. The qualitative differences in the intensity distributions are probably driven by the biological differences between the regions that the two probe types are interrogating, which is reflected by the difference in density of CpGs in the body of the probes.

Normalization is intended to remove sources of technical variation between measurements. In this process, however, it is important that any true biological differences between samples and probes are maintained. It has been observed that there are differences between Infinium I and Infinium II probes that are clearly technical in nature [21,26]. However, true biological differences also exist between the probe types due to the design criteria of the array. Figure 2 shows the differences in the intensity distributions of the Infinium I and Infinium II probes. We assume that most of the qualitative differences can be explained by the fact that, on average, the Infinium I probes contain a higher proportion of CpGs along the body of the probe. Specifically, probes with a high proportion of CpGs are more likely to be in CpG dense areas of the genome and therefore often reside in CpG islands, while probes with few CpGs are less likely to be in CpG islands. Based on the Illumina annotation, 57% of Infinium I probes are found in CpG islands, whilst only 21% of Infinium II probes are designated as islands. It has been well documented that CpGs in CpG islands have different methylation patterns compared to CpGs in the rest of the genome [27-29]. Therefore, it is not surprising that the distributions of the intensities of Infinium I and Infinium II probes are vastly different.

Because the two probe types interrogate different subsets of the genome, established methods for normalization, such as quantile normalization, cannot be applied naively between probe types. Standard quantile normalization makes the distribution of probe intensities for each array in a set of arrays identical. More recently, a subset quantile normalization approach was introduced that uses large sets of control probes on the arrays for normalization and assumes that only the distributions of these control probes remain constant [30]. However, there are no large sets of controls that have probes corresponding to both the Infinium I and Infinium II designs on the 450k platform.

**Figure 3:**
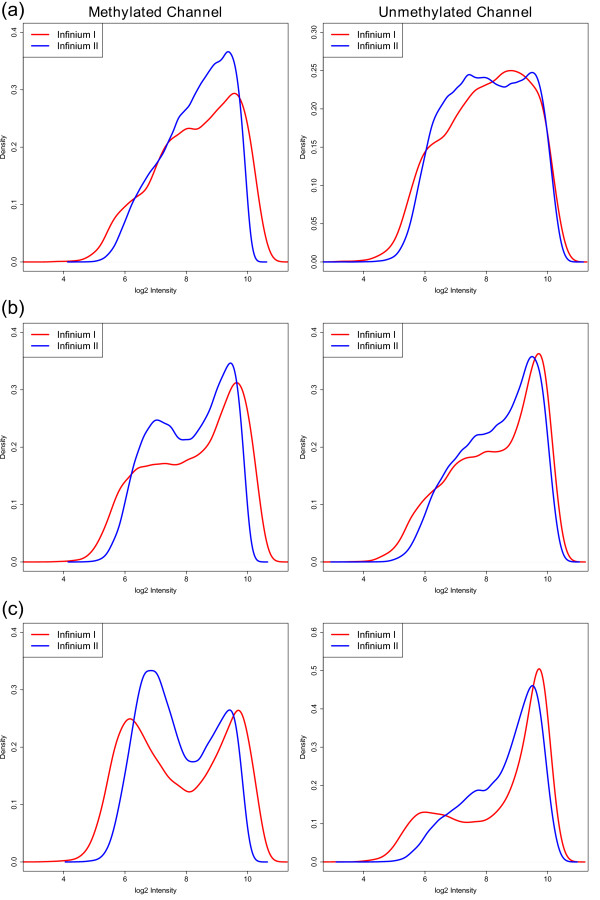
**Intensity distributions of subsets of Infinium I and II probes with the same number of underlying CpGs for normal human kidney sample TCGA-B0-5092-11**. **(a) **One CpG in the probe body. **(b) **Two CpGs in the probe body. **(c) **Three CpGs in the probe body.

**Figure 4:**
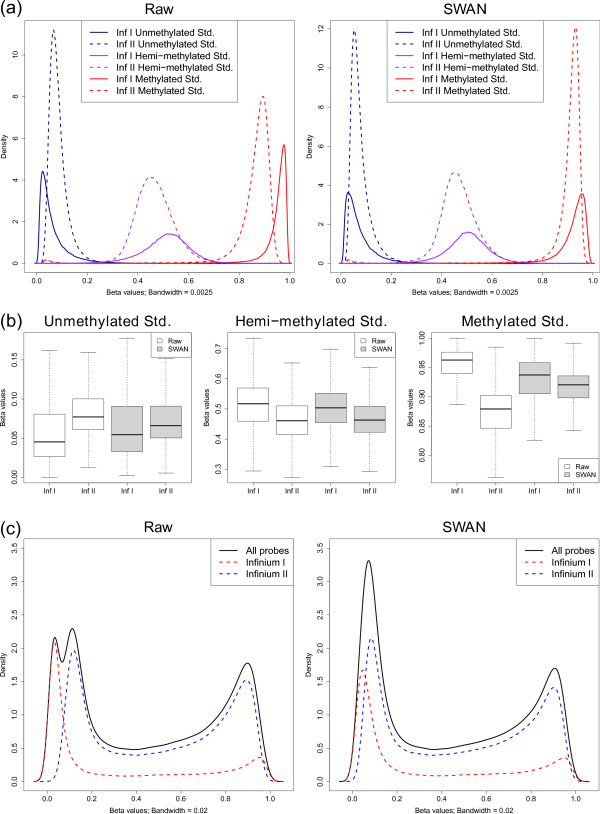
**Differences in β value distributions produced by Infinium I and II probes**. **(a) **Density distributions of β values produced by Infinium I (solid line) and II (dashed line) probes for unmethylated (blue), hemi-methylated (purple) and methylated (red) reference standards (Std.). The difference in β value distribution between the Infinium I and II probes seen in the raw data can be adjusted for using the SWAN method. **(b) **The median and inter-quartile range of β value distributions for Infinium I and II probes is more similar when SWAN is applied to 450k data. **(c) **The differences in β value distributions produced by the different probe types can result in aberrant overall β value distributions, as seen in this normal human DNA sample. Applying the SWAN method results in an improvement in the overall β value distribution.

In order to develop a normalization method, we assume that the overall intensity distribution should be the same when the underlying CpG contents of the probes are the same. In other words, we assume the CpG content of the probes reflects the biology by being a surrogate for the CpG density of the region. Indeed, we find that the intensity distributions of probes with the same number of CpGs in the probe body are similar (Figure 3). The degree of similarity does vary on a sample to sample basis; however, we generally find that the intensity distributions of probes with the same number of CpGs in the probe body are more similar than the overall intensity distributions. Q-Q plots (Figure S1 in Additional file 1) of the sample shown in Figure 4 further support our observation, as they appear to be more linear when probes are grouped by their underlying CpG content than when all probes are considered together. Hence, the underlying assumption in our proposed method is that the differences in the intensity distributions between the probe types, seen in Figure 4, represent technical differences between the Infinium I and II probe types.

The SWAN method has two parts. First, an average quantile distribution is determined using a subset of probes defined to be biologically similar based on CpG content. This is achieved by randomly selecting *N *Infinium I and II probes that have one, two and three underlying CpGs, where *N *is the minimum number of probes in the six sets of Infinium I and II probes with one, two and three probe body CpGs. If no probes have been filtered out - for example, sex chromosome probes, and so on - *N *= 11,303. This results in a pool of 3*N *Infinium I and 3*N *Infinium II probes. Due to the vast differences in their distributions (Figure 2), the subsequent processing is performed independently on both the methylated and unmethylated channels. The subset for each probe type, from each channel (methylated or unmethylated), is sorted by increasing intensity. The value of each of the 3*N *pairs of observations is then assigned to be the mean intensity of the two probe types for that row or 'quantile'. This is the standard quantile procedure. The second step is to then adjust the intensities of the remaining probes, of which there are many more Infinium II than I, by interpolation onto the distribution of the subset probes. This is done for each probe type separately using linear interpolation between the subset probes to define the new intensities. Consequently, while the distribution of the subset is identical, the intensity distribution of Infinium I probes is still vastly different from the distribution of Infinium II probes (Figure S2 in Additional file 1).

### SWAN makes Infinium I and II β value distributions more similar

We applied the SWAN method to the fully methylated (FM), fully unmethylated (FU) and hemi-methylated (HM) sample analyzed by Bibikova *et al*. [20]. The raw data were imported from IDAT files using the *minfi *package [25]. SWAN was applied to the raw intensity data and β values were calculated using the methylated and unmethylated intensity values for both the raw and SWAN normalized data. Figure 4a shows the raw and SWAN normalized β value distributions for Infinium I and II probes for all three methylation standards. It can be seen that after SWAN the average β value distributions from the two probe types are more consistent, particularly for the FM and FU samples. Furthermore, the absolute difference in the medians of the Infinium I and II β value distributions is reduced after using SWAN for all three standards (Figure 4b; difference in medians: FM-Raw: 0.032, FM-SWAN: 0.012; HM-Raw: 0.057, HM-SWAN: 0.041; FU-Raw: 0.084, FU-SWAN: 0.017). Figure 4c shows an example of how the differences in the two probe types can result in an aberrant overall β value distribution for a normal human DNA sample. Using SWAN, however, corrects the overall distribution (Figure 4c). The SWAN procedure reduces the absolute difference between the peak positions of Infinium I and Infinium II probes at the unmethylated (ΔP_U_) and methylated (ΔP_M_) extremes of the distribution (see Materials and methods). For the data shown in Figure 4c, ΔP_U _is reduced from 0.067 to 0.046, whilst ΔP_M _remains relatively unchanged from 0.035 to 0.038, resulting in an improved overall β value distribution. Although the changes to the overall β value distribution appear dramatic for some samples, not all samples have large differences in probe type distributions. Usually the changes to the β values of individual CpGs after SWAN are less than ±0.1 (Figure S3 in Additional file 1).

Next we compared the results of methylation analysis for an MCF7 sample that was assayed on the 450k array and the older 27k array. The 27k array only includes probes of the Infinium I design. Of the CpGs interrogated on the 27k array, 25,978 are included on the 450k array but many (91%) of them are now assayed using the Infinium II design, while the remaining sites are still assayed using the Infinium I design. We found that using SWAN made the 450k Infinium I and II β value distributions more similar to those of 27k by reducing the absolute difference in the locations of the peaks at the extremes of the distribution (Figure 6). ΔP_U _is reduced for 27k compared to 450k Infinium I from 0.021 to 0.018 and Infinium II from 0.0174 to 0.0167, whilst ΔP_M _is also reduced for 27k compared to 450k Infinium I from 0.085 to 0.067 and Infinium II 0.04 to 0.013.

### SWAN reduces technical variability

**Figure 5:**
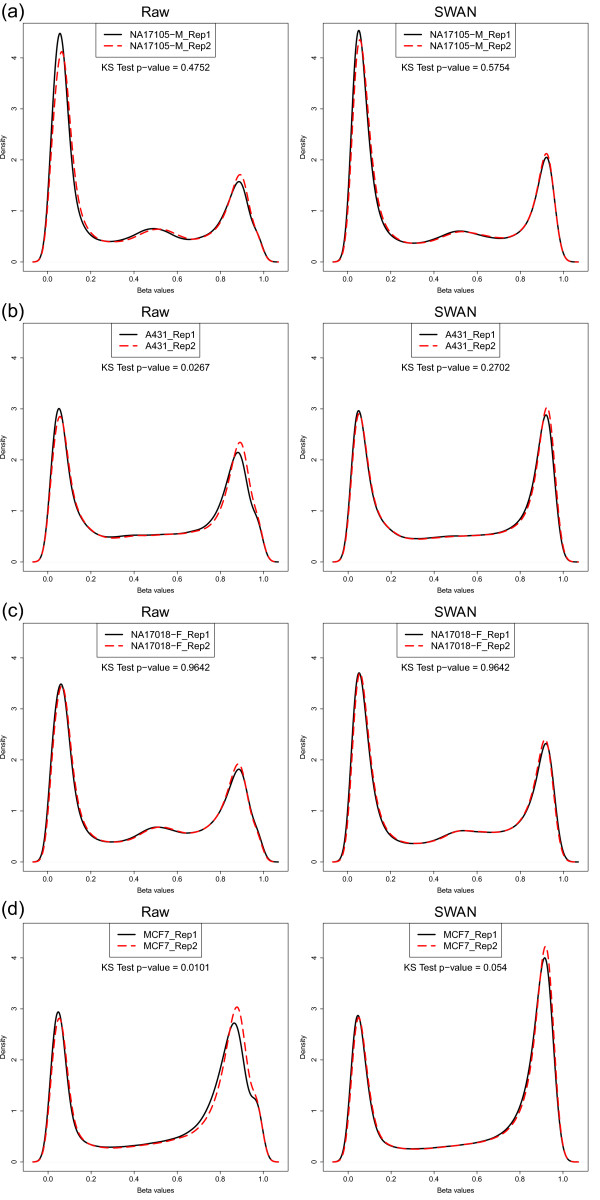
**β value density distributions for four pairs of technical replicates before and after applying SWAN**. **(a-d) **β value density distributions for each pair of technical replicates. The Kolmogorov-Smirnov (KS) test *P*-value reflects the similarity of the β value distributions between each pair of replicates; a larger *P*-value indicates that the distributions of the replicates are more similar.

Next, we used four sets of technical replicates to show that SWAN reduces overall technical variability between arrays. The DNA samples, NA17105 and NA17018, and the MCF7 and A431 cancer cell lines were originally reported in Bibikova *et al*. [20] as technical replicates. We compared the density distributions of β values between the technical replicates for both the raw and SWAN normalized data (Figure 5). The similarity between the β value distributions of each pair of replicates was tested using the Kolmogorov-Smirnov (KS) test. The null hypothesis of the KS test is that the two replicates have the same distribution; therefore, a larger *P*-value indicates greater similarity between the distributions. The results of the KS test for each set of replicates indicate that the β value distributions of the replicates are more similar after using SWAN. The same result was obtained when we performed the KS test on the M-value (log_2_(Methylated/Unmethylated)) distributions of the replicate pairs (Figure S4 in Additional file 1). Furthermore, the correlation between replicates, although high to begin with, always increased (Figure S5 in Additional file 1). Together, this indicates that although SWAN is a within array procedure that makes the Infinium I and Infinium II probes comparable, it also reduces technical variability when comparing between arrays by accounting for technical differences in the comparison of the two probe types between arrays. In other words, the difference between the distributions of Infinium I and Infinium II probes varies on an array by array basis regardless of the sample that is hybridized.

### SWAN leads to better detection of differential methylation

We have shown that SWAN reduces technical variation between arrays. In order to show that the biological differences of interest have been maintained, we performed a differential methylation analysis between two groups, as reducing technical variation whilst maintaining biological differences should increase the power to detect truly differentially methylated CpGs. We performed a differential methylation analysis comparing three normal human kidney samples to three normal human rectum samples. To evaluate the impact of SWAN on differential methylation analysis, unrelated kidney and rectal mucosa samples analyzed by reduced-representation bisulfite sequencing (RRBS) were used to define a set of 'truly' differentially methylated loci (see Materials and methods).

All of the processing and analysis of the 450k data were performed using the *minfi *Bioconductor package [25] (see Materials and methods). As there was a mixture of male and female donors, probes on the × and Y chromosomes were excluded prior to further analysis. The data were then normalized using SWAN. Probes with a detection *P*-value >0.01 in at least one sample were removed at this stage from both the raw and SWAN treated data. A subset of 18,678 CpGs that overlapped with the RRBS methylation data was selected for differential analysis. This subset contained approximately equal numbers of Infinium I (44.6%) and Infinium II (55.4%) probes. An identical differential methylation analysis was performed using the testing available in *minfi *on both the raw and SWAN treated data. Figure 7a shows that a higher percentage of true positives was identified using SWAN at a range of q-value thresholds. Furthermore, the receiver operating characteristic (ROC) curve [31], seen in Figure 7b, demonstrates that the analysis using SWAN consistently outperformed the analysis of the raw data. These results indicate that whilst SWAN reduces technical variation, it does not reduce sensitivity.

**Figure 6:**
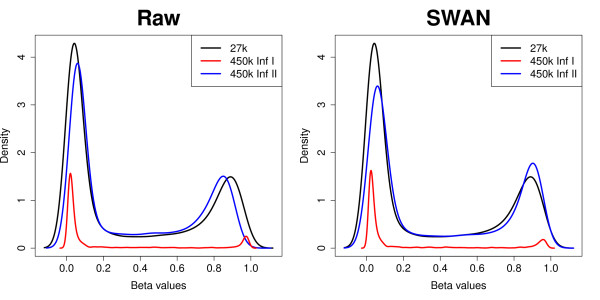
**Comparison of β value density distributions between HumanMethylation450 and HumanMethylation27 arrays before and after SWAN in an MCF7 cell line**. This plot illustrates the β value density distributions for 25,978 CpGs that are present on both 450k and 27k platforms. The peaks of the 450k Infinium I and II probe types show better alignment with the 27k peaks when SWAN is used.

**Figure 7:**
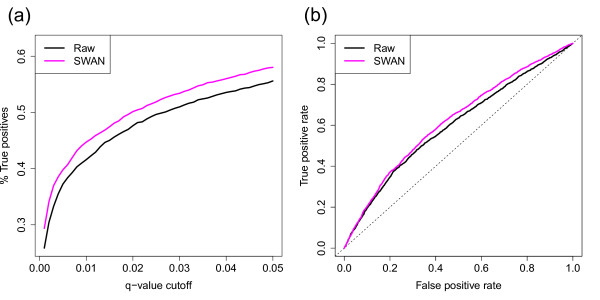
**Results of differential methylation analysis of three kidney samples compared to three rectum samples, with and without using SWAN**. **(a) **Percentage of RRBS true positives identified at various q-value significance thresholds. Using the SWAN method (magenta) consistently detects more RRBS true positives than analyzing raw data (black). **(b) **Receiver operating characteristic (ROC) curve of an analysis using SWAN compared to an analysis of the raw data. Using SWAN (magenta) prior to differential methylation analysis results in performance gains.

We performed a second differential methylation analysis comparing three male arrays with two female arrays where each array is a pool of two individuals. The data were processed using three different methods: no normalization (raw), Illumina's control normalization as implemented in *minfi *and SWAN. Probes on the Y chromosome and probes with a detection *P*-value >0.01 in one or more samples were excluded, leaving a total of 482,704 probes for differential methylation analysis. Differential methylation analysis was performed using the testing available in *minfi *on each of the three different versions of the data. Using SWAN consistently resulted in a higher number of significantly differentially methylated probes (DMPs) across a range of q-value thresholds (Figure S6a in Additional file 1). Furthermore, using SWAN facilitated the detection of more unique DMPs (170) than using the other methods (118) (Figures S6b and S7 in Additional file 1). We also found that, as expected, a larger proportion (77.6%) of the unique DMPs detected when using SWAN was from the × chromosome, when compared with the combined set of unique DMPs detected using the other methods (63.6%).

## Discussion

The HumanMethylation450 BeadChip includes a combination of two different probe designs for assaying the methylation status of 485,577 CpG sites across the human genome. This unique design clearly produces technical differences between probe types within a single array. Here we present a new within array normalization method that substantially reduces the technical variability between the probe types whilst maintaining the important biological differences.

The SWAN method makes the assumption that the number of CpGs within the 50 bp probe sequence reflects the underlying biology of the region being interrogated. Hence, the overall distribution of intensities of probes with the same number of CpGs in the probe body should be the same. The method then uses a subset quantile normalization approach to adjust the intensities of the probes on the arrays.

SWAN clearly improves the results obtained from the 450k array. We show that technical variability is reduced, whilst increasing the ability to detect differential methylation between samples. We also report better correlation between the 450k arrays and the 27k arrays, which will be important for studies that aim to combine data from both platforms.

Although further investigations into other aspects of the analysis of these arrays, such as color normalization, between array normalization and statistical testing procedures for differential methylation may prove beneficial, we feel that SWAN is an essential step in the analysis of the Illumina Infinium HumanMethylation450 BeadChip. SWAN is available in the Bioconductor package *minfi *[25].

## Materials and methods

### Data

The HumanMethylation450 data for the unmethylated, methylated and hemi-methylated reference standards, as well as the NA17105 and NA17018 DNA samples, and the MCF7 and A431 cancer cell lines were obtained from the Illumina website in the raw IDAT format. The HumanMethylation27 MCF7 data were kindly provided by Dr Marina Bibikova (Illumina).

The normal human kidney and rectum methylation data were sourced from The Cancer Genome Atlas (TCGA) Data Portal [32]. Specifically, the normal kidney samples (TCGA-B0-5121-11, TCGA-BP-4177-11 and TCGA-B0-5092-11) were part of the kidney renal clear cell carcinoma cohort, whilst the normal rectum samples (TCGA-AG-3731-11, TCGA-AG-3725-11 and TCGA-B0-5121-11) were from the rectal adenocarcinoma cohort. All the data were in the raw IDAT format.

The RRBS data were obtained from the Epigenomics Roadmap at NCBI [33]. The normal human kidney (NA000003582.1) and normal human primary rectal mucosal tissue (NA000003579.1) samples were both obtained in WIG format, which is a series of base pair positions with corresponding β values for each chromosome.

The data for the male versus female differential methylation comparison comprise a subset of data generated for an unrelated study by Martino *et al*. [34]. Briefly, the five HumanMethylation450 arrays used in this study were hybridized with bisulfite converted DNA pooled from three samples from two male individuals and two samples from two female individuals extracted from mononuclear cells collected at birth. These data were also in the raw IDAT format.

### Normalization

As described in the results, the SWAN method has two parts. An average quantile distribution is firstly determined using a randomly selected subset of probes defined to be biologically similar based on CpG content. The subset for each probe type, from the methylated and unmethylated channels separately, is then sorted by increasing intensity and the value of each observation is assigned to be the mean intensity of the two probe types for that row or 'quantile'. Subsequently, the intensities of the remaining probes are adjusted for each probe type separately using linear interpolation between the subset probes to define the new intensities. However, if probe *i *has an intensity greater than the maximum intensity of the subset probes, then it is given an intensity using the following rule:

$$\begin{array}{c} x_{i}>\text{max}\left(x_{sub}\right)\\ d_{i}=x_{i}-\text{max}\left(x_{sub}\right)\\ x_{i}^{\prime }=\text{max}\left(x_{sub}^{\prime }\right)+d_{i} \end{array}$$

where *x *are the measured intensities and *x*' are the normalized intensities for probe *i*, and the normalization subset is denoted by *sub*. Similarly, for probes with intensities less than the minimum intensity in the subset, the rule is:

$$\begin{array}{c} x_{i}<\text{min}\left(x_{sub}\right)\\ d_{i}=\text{min}\left(x_{sub}\right)-x_{i}\\ x_{i}^{\prime }=\text{min}\left(x_{sub}^{\prime }\right)-d_{i} \end{array}$$

If the normalized intensity of any probe is less than or equal to zero, its intensity is set to the median intensity of the negative control probes.

### Calculating ΔP

In order to assess the performance of the method, we calculated the difference in the peak positions of the Infinium I and Infinium II probes [21]. We define P_U _to be the position of the maximum of the β distribution with β < 0.5 (unmethylated peak) and P_M _to be the position of the maximum with β > 0.5 (methylated peak). We define the absolute difference in peak positions between Infinium I and Infinium II probes as |ΔP = P_I _- P_II_| for both the methylated and unmethylated peaks.

### Selecting RRBS validation data

To identify CpG loci that were interrogated in both the RRBS and HumanMethylation450 data, we firstly identified a set of CpGs that were assayed in both the kidney and rectum RRBS samples. The resulting list was then intersected with the probe locations of the HumanMethylation450 data. This produced a subset of 18,678 CpG loci.

### Differential methylation analysis: tissue comparison

IDAT files were loaded into the R (2.14) environment using the Bioconductor (2.9) *minfi *package (1.0.0) [25]. The detection *P*-values for all probes were then calculated for the data using functionality provided in *minfi*. Probes on the × and Y chromosomes were removed at this stage. Two versions of the data were used in subsequent analyses: the raw data and SWAN data. Probes with a detection *P*-value >0.01 in one or more samples were then excluded. The differential methylation analysis was performed for both datasets on the subset of 18,678 probes that overlapped with the RRBS data using the 'dmpFinder' *minfi *function. The 'dmpFinder' function uses an F-test to identify positions that are differentially methylated between two groups. The tests are performed on M-values (log_2_(Methylated/Unmethylated)) as recommended in Du *et al*. [35]. Variance shrinkage was used due to the small sample size. In 'dmpFinder', the sample variances are squeezed by computing empirical Bayes posterior means using the limma package [36]. Example R code for performing a differential methylation analysis using *minfi *can be found in Additional file 2.

True positives were defined to be CpGs that had an absolute difference in β value >0.25 between the kidney and rectum RRBS samples. Additionally, for the ROC analysis, which was performed using the ROCR package [31], true negatives were defined as those CpGs found to have an absolute difference in β value <0.05 between the RRBS samples.

## Abbreviations

27k: HumanMethylation27; 450k: HumanMethylation450; bp: base pair; DMP: differentially methylated probe; FM: fully methylated; FU: fully unmethylated; HM: hemi-methylated; KS: Kolmogorov-Smirnov; ROC: receiver operating characteristic; RRBS: reduced representation bisulfite sequencing; SWAN: Subset-quantile Within Array Normalization; TCGA: The Cancer Genome Atlas.

## Competing interests

The authors declare that they have no competing interests.

## Authors' contributions

AO conceived the idea. JM performed the analysis and wrote the code. LG contributed to data exploration and discussion. AO and JM wrote the paper. All authors read, edited and approved the final manuscript.

## Supplementary Material

Additional file 1 **Supplementary Figures S1 to S7**. Click here for file

Additional file 2 **Example differential methylation analysis using *minfi *and SWAN in R**. Click here for file
