# Supplementary material for: SWAN: Subset-quantile Within Array Normalization for Illumina Infinium HumanMethylation450 BeadChips
Source: Genome Biol. 2012 Jun 15;13(6):R44. doi: 10.1186/gb-2012-13-6-r44 (PMC3446316; doi:10.1186/gb-2012-13-6-r44)
Supplement: Additional file 1 — Supplementary Figures S1 to S7. [file gb-2012-13-6-r44-S1.PDF]

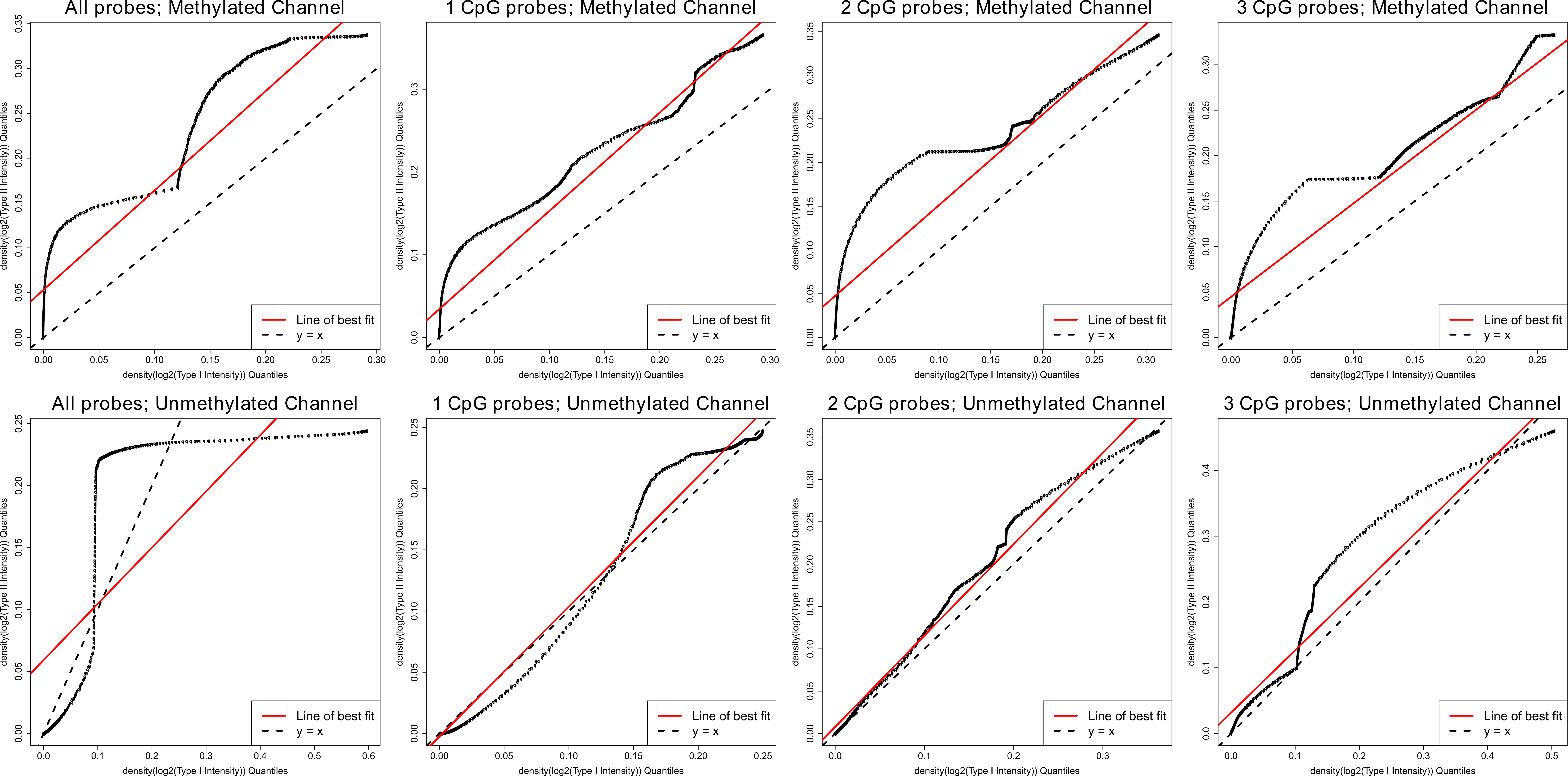

Supplementary Figure 1. Q-Q Plots of the intensity distributions of Infinium I and II probes for sample TCGA−B0−5092−11. The methylated (top) and unmethylated (bottom) channels are shown separately. If the two distributions being compared are similar, the points in the Q-Q plot will approximately lie on the line y = x. If the distributions are linearly related, the points in the Q-Q plot will approximately lie on a line, but not necessarily on the line y = x. The top and bottom panels on the far left describe the relationship of the distributions of the Infinium I and II probes when all probes are considered together. The remaining panels describe the relationship of the Infinium I and II distributions when only probes with the same number of body CpGs are considered together. The distributions of the Infinium I and II probes appear to be more similar when grouped by the number of probe body CpGs.

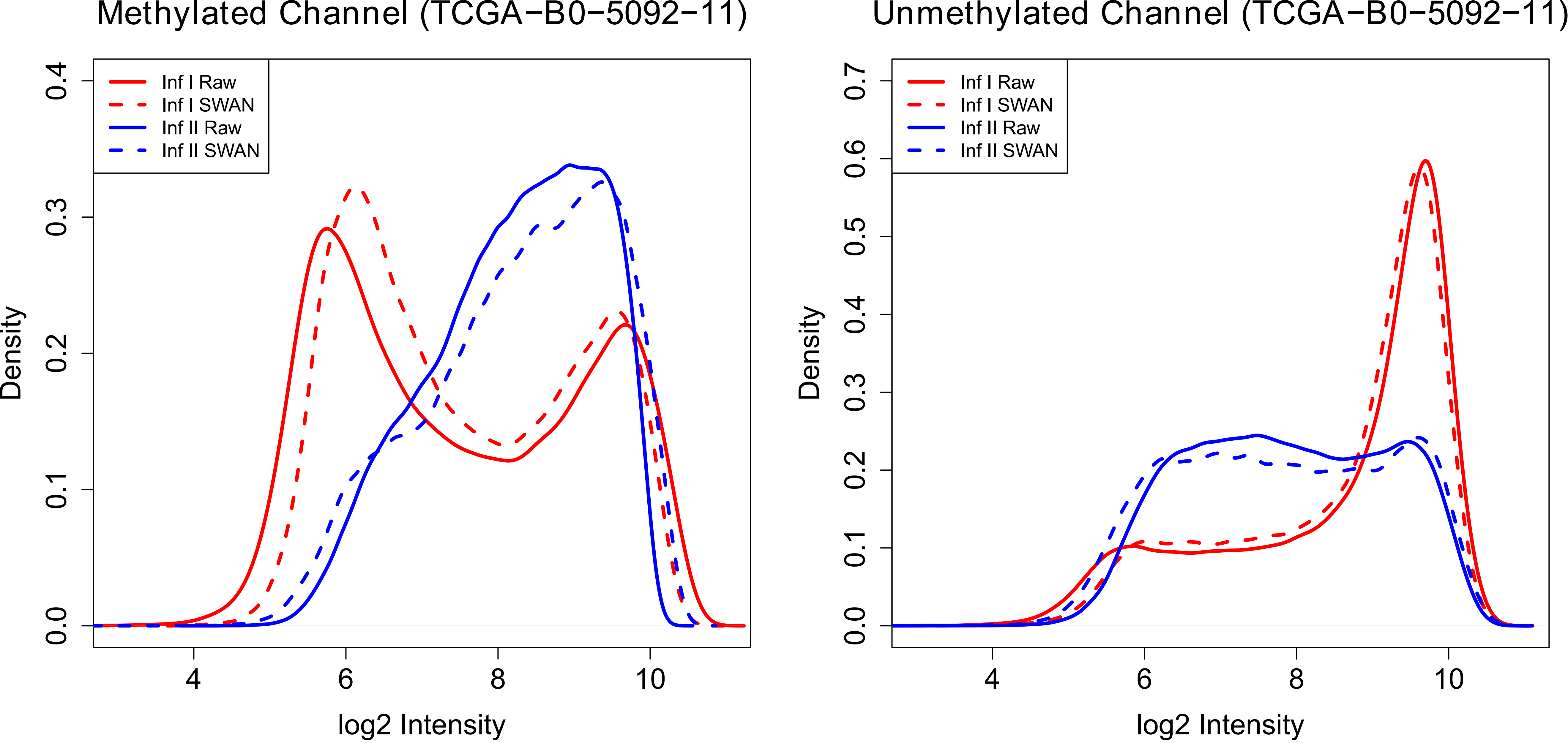

Supplementary Figure 2. Intensity distributions of Infinium I and II probes before and after SWAN. The the methylated (top) and unmethylated (bottom) channels are shown separately. The distributions of the Infinium I (red) and Infinium II (blue) probes are vastly different before (solid line) and after (dashed line) SWAN.

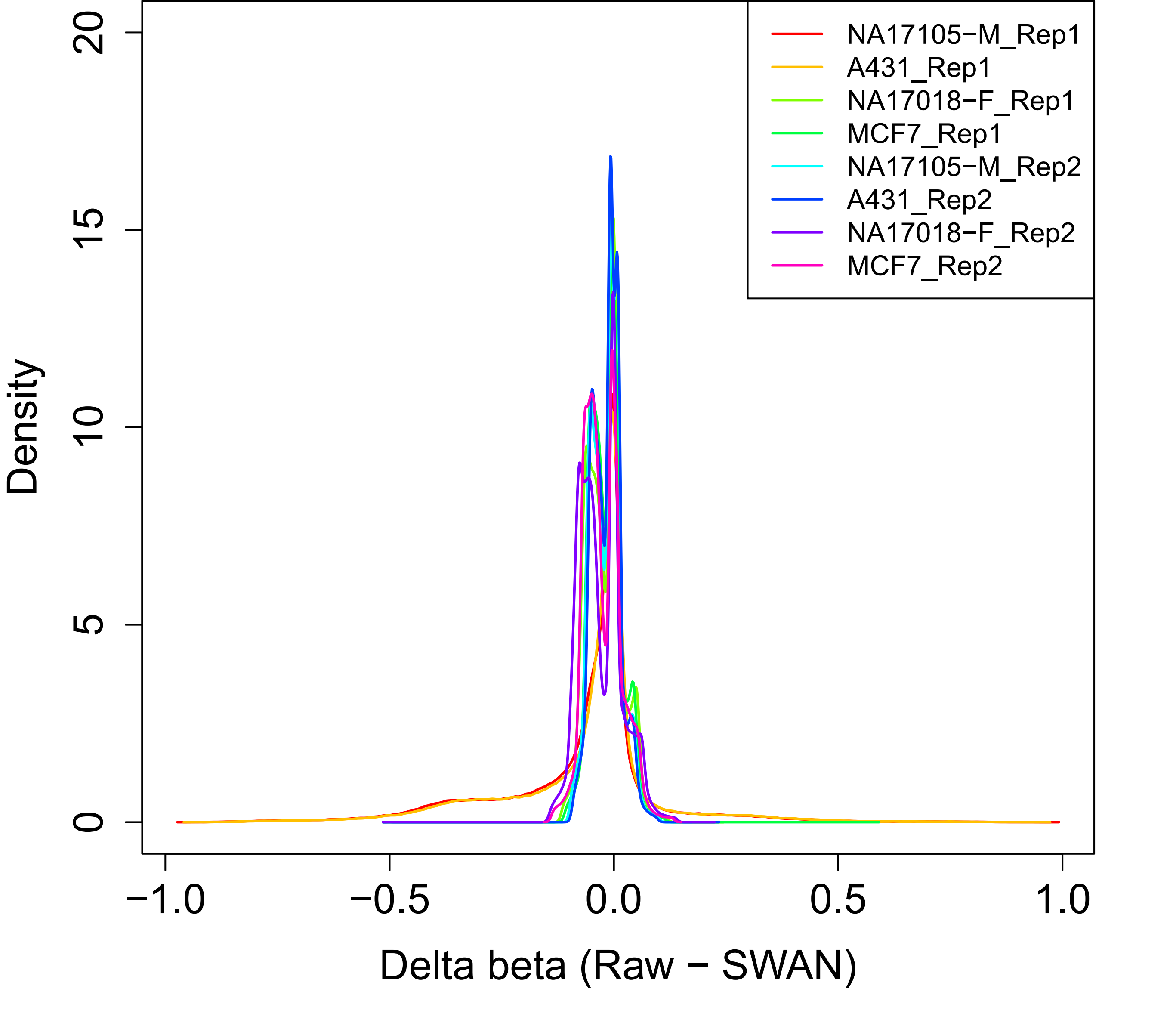

Supplementary Figure 3. Changes in beta values for individual CpGs when SWAN is used across a variety of samples. The beta values for most CpGs change by less than |0.1|.

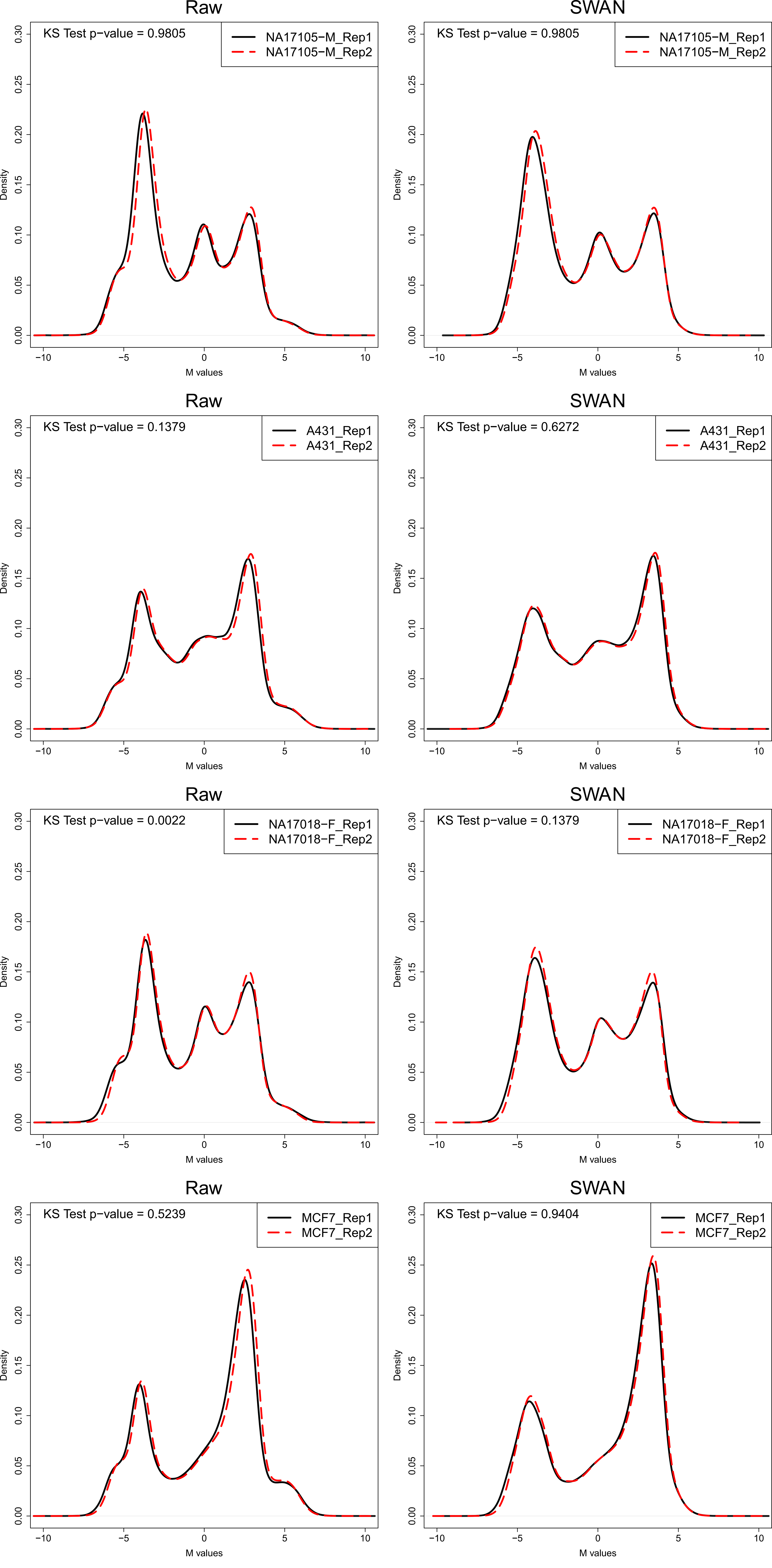

Supplementary Figure 4. M-value density distributions of technical replicated before and after SWAN. The KS (Kolmogorov-Smirnov) Test p-value reflects the similarity of the β value distributions between each pair of replicates; a larger the p-value indicates that the distributions of the replicates are more similar.

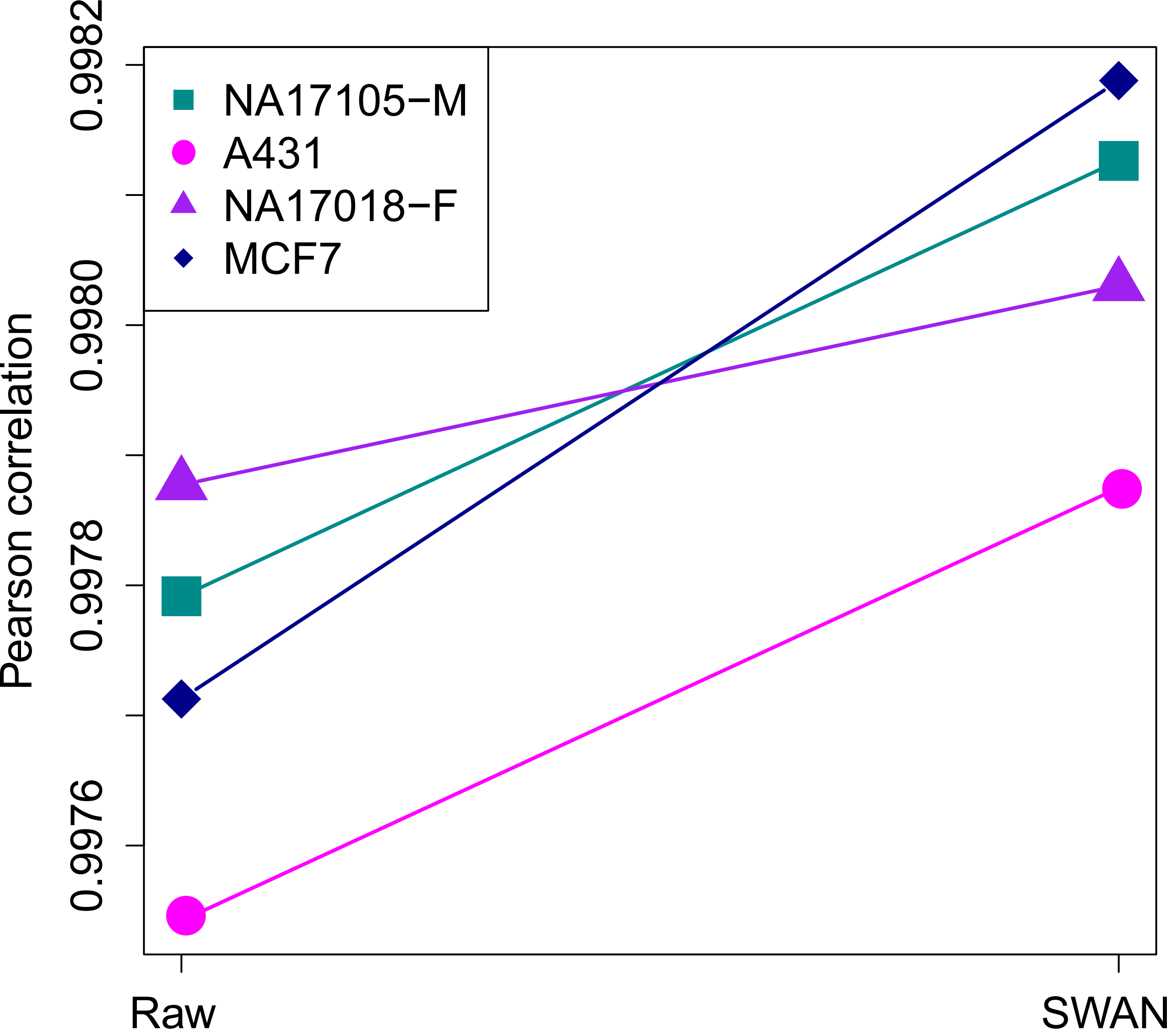

Supplementary Figure 5. Correlation between 4 pairs of technical replicates before and after SWAN. The Pearson correlation coefficient is highest for each pair of technical replicates after using SWAN.

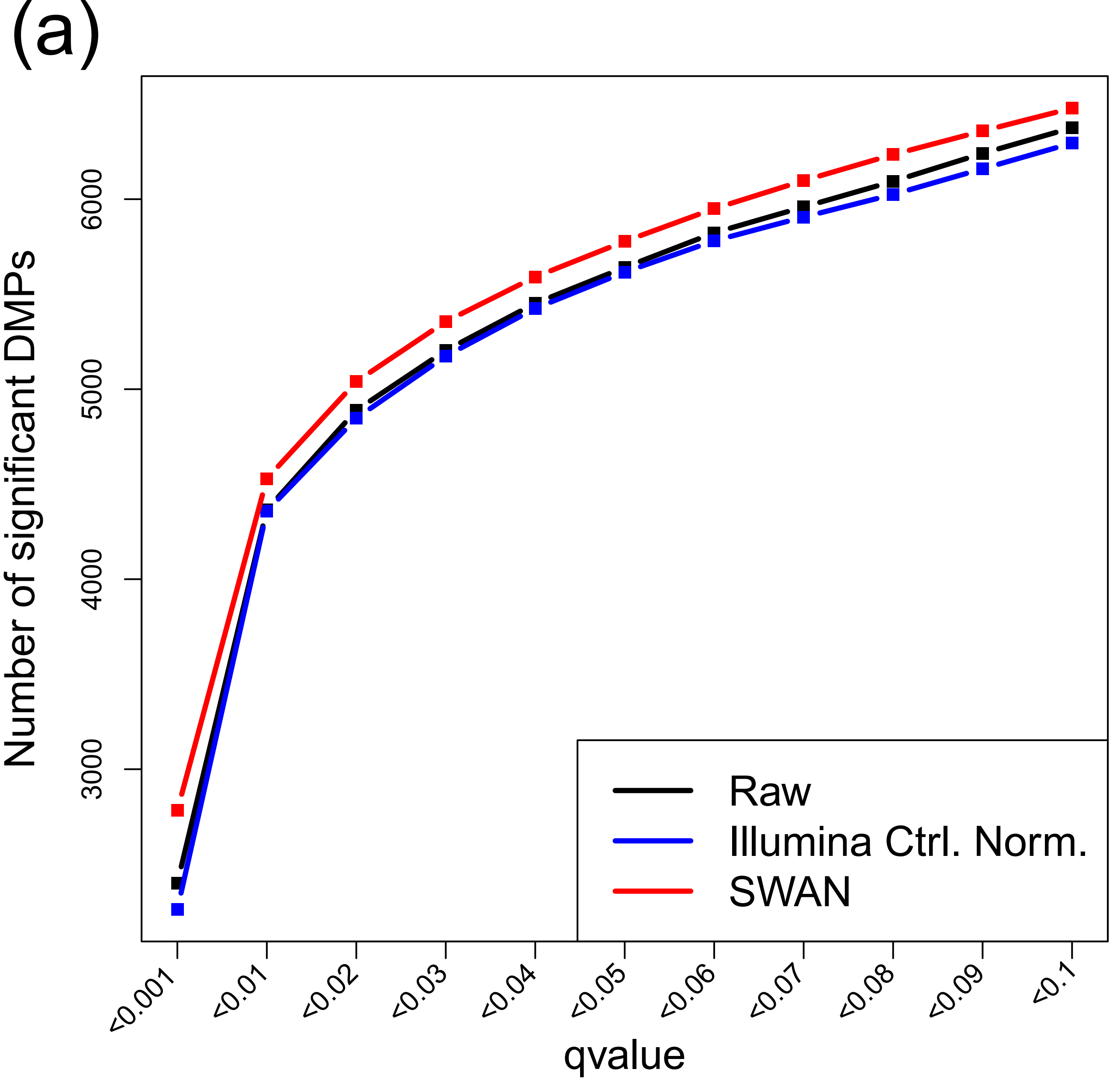

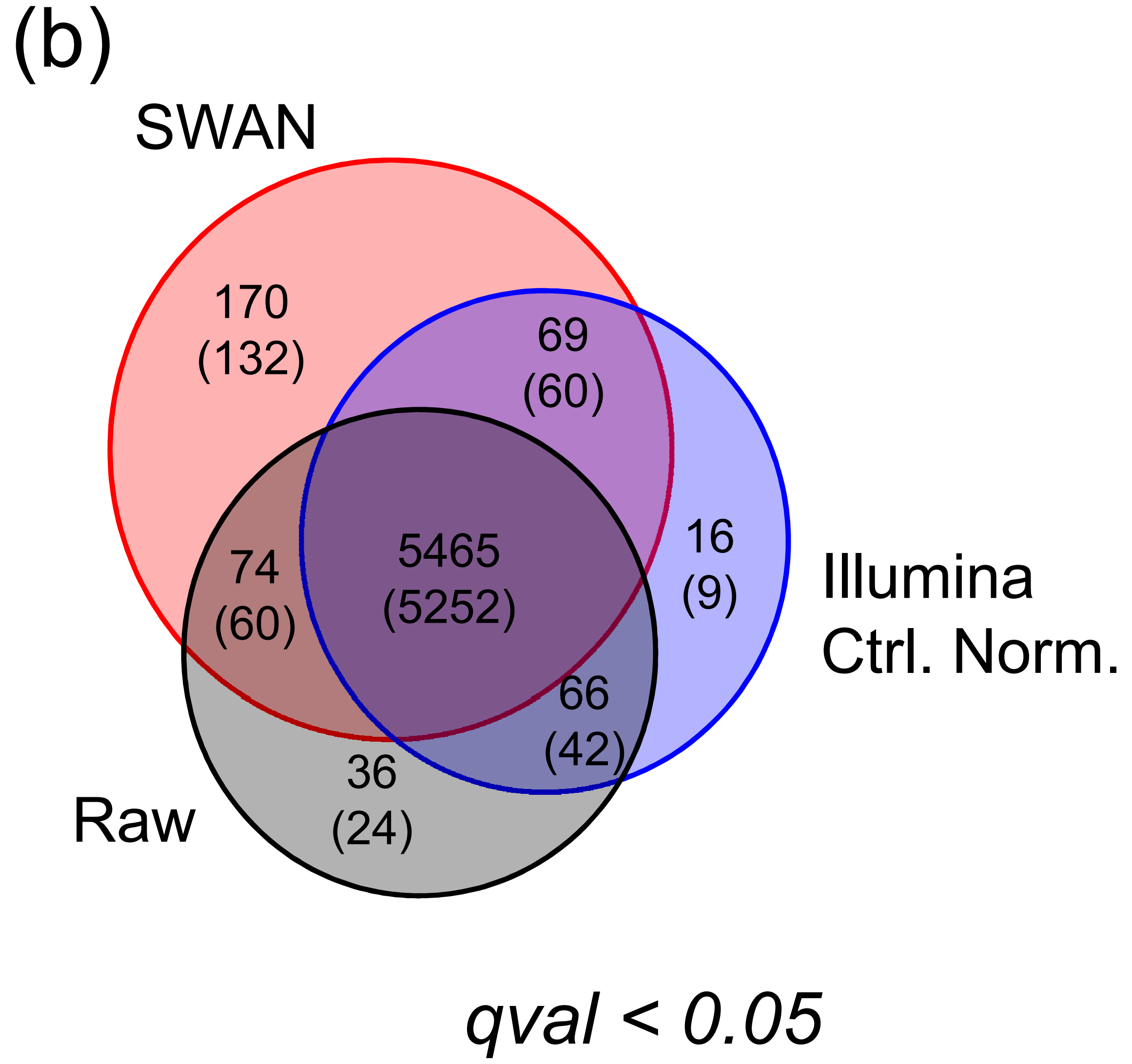

Supplementary Figure 6. Results of differential methylation analysis of 3 males compared to 2 females with and without using SWAN. (a) Number of significantly differentially methylated probes (DMPs) at various qvalue significance thresholds. Performing Illumina's control probe normalization as implemented in mifi (blue) only produces more significant DMPs than analysing raw data only at the lowest qvalue cut offs. Using the SWAN method (red) consistently yields more significant DMPs than the other methods. (b) Number of significant DMPs obtained when comparing male and female samples (qvalue < 0.05), after applying different normalization strategies. The number of significant DMPs on the X chromosome is indicated in brackets. Using the SWAN method results in the detection of more unique DMPs, including more X chromosome probes, than using the other methods.

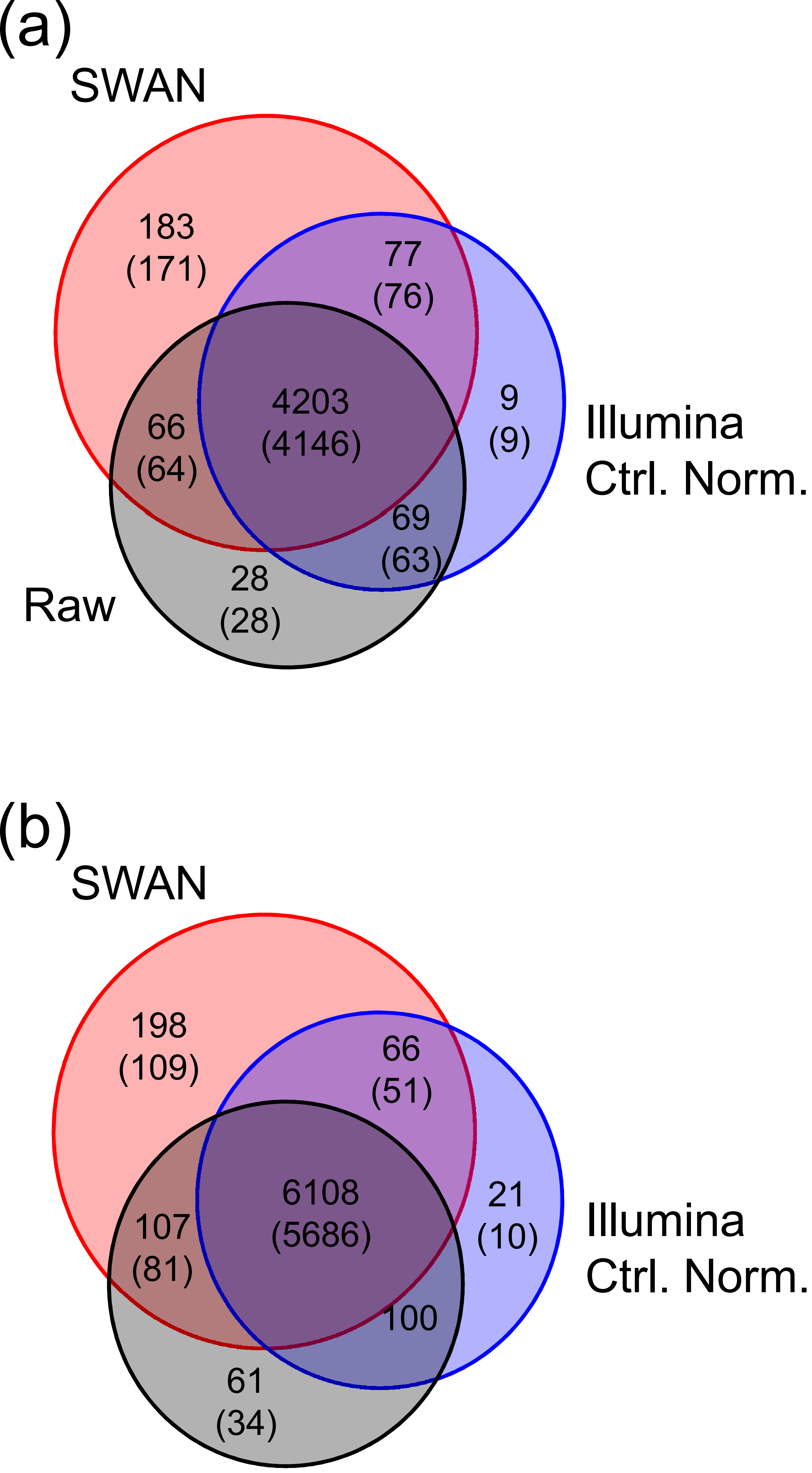

Supplementary Figure 7. Number of significant DMPs obtained when comparing male and female samples after applying different normalization strategies. (a) Significance cutoff: qvalue < 0.01. (b) Significance cutoff: qvalue < 0.10. The number of significant DMPs on the X chromosome is indicated in brackets. Using the SWAN method results in the detection of more unique DMPs, including more X chromosome probes, than using the other methods.
